# Pancreatic cancers depend on a non-canonical glutamine metabolism pathway

**DOI:** 10.1186/2049-3002-2-S1-P44

**Published:** 2014-05-28

**Authors:** Costas Lyssiotis, Jaekyoung Son, Joesph Mancias, Haoqiang Ying, Alec Kimmelman, Lewis Cantley

**Affiliations:** 1 Weill Cornell Medical College, New York, NY, USA; 2 Dana-Farber Cancer Institute, Boston, MA, USA; 3 MD Anderson Cancer Center, Houston, TX, USA

## 

Cancer cells exhibit metabolic dependencies that distinguish them from their normal counterparts. Among these addictions is an increased utilization of the amino acid glutamine (Gln) to fuel anabolic processes. Recently, we reported the identification of a non-canonical pathway of Gln utilization in human pancreatic cancer cells that is required for tumor growth. While most cells utilize glutamate dehydrogenase (GLUD1) to convert Gln-derived glutamate (Glu) into α-ketoglutarate (αKG) in the mitochondria to fuel the tricarboxylic acid cycle, pancreatic cancer cells rely on a distinct pathway that integrates the mitochondrial and cytosolic aspartate aminotransferases GOT2 and GOT1. By generating αKG from Glu (in conjunction with the conversion of oxaloacetate into aspartate), GOT2 fuels anaplerosis in place of GLUD1. The Asp created is released into the cytosol and acted on by GOT1. This is subsequently used through a series of reactions to yield cytosolic NADPH from malic enzyme. Importantly, we have demonstrated that pancreatic cancers are strongly dependent on this series of reactions to maintain redox homeostasis which enables proliferation. Herein, we detail the subcellular compartmentalization and consequences of the aforementioned reactions on pancreatic cancer metabolism. We have also investigated the essentiality of this pathway in other contexts and find that pancreatic cancers have a uniform and unique reliance on this pathway, which may provide novel therapeutic approaches to treat these refractory tumors.

